# Maximum thermal tolerance trades off with chronic tolerance of high temperature in contrasting thermal populations of *Radix balthica*

**DOI:** 10.1002/ece3.2923

**Published:** 2017-03-30

**Authors:** Magnus P. Johansson, Anssi Laurila

**Affiliations:** ^1^ Animal Ecology/Department of Ecology and GeneticsEvolutionary Biology CentreUppsala UniversityUppsalaSweden

**Keywords:** acute thermal stress, chronic thermal stress, critical thermal maximum, gastropods, gene flow, geothermal spring, Lake Mývatn, preferred temperature, thermal adaptation

## Abstract

Thermal adaptation theory predicts that thermal specialists evolve in environments with low temporal and high spatial thermal variation, whereas thermal generalists are favored in environments with high temporal and low spatial variation. The thermal environment of many organisms is predicted to change with globally increasing temperatures and thermal specialists are presumably at higher risk than thermal generalists. Here we investigated critical thermal maximum (CT_max_) and preferred temperature (*T*_p_) in populations of the common pond snail (*Radix balthica*) originating from a small‐scale system of geothermal springs in northern Iceland, where stable cold (ca. 7°C) and warm (ca. 23°C) habitats are connected with habitats following the seasonal thermal variation. Irrespective of thermal origin, we found a common *T*_p_ for all populations, corresponding to the common temperature optimum (*T*_opt_) for fitness‐related traits in these populations. Warm‐origin snails had lowest CT_max_. As our previous studies have found higher chronic temperature tolerance in the warm populations, we suggest that there is a trade‐off between high temperature tolerance and performance in other fitness components, including tolerance to chronic thermal stress. *T*_p_ and CT_max_ were positively correlated in warm‐origin snails, suggesting a need to maintain a minimum “warming tolerance” (difference in CT_max_ and habitat temperature) in warm environments. Our results highlight the importance of high mean temperature in shaping thermal performance curves.

## 1 Introduction

Temperature is a ubiquitous force influencing most biological processes in nature (Angilletta, 2009; Kingsolver & Huey, 2008). With globally increasing temperatures (IPCC, 2013), the need to understand thermal performance and how organisms can adapt to changing temperature regimes has become more urgent (Angilletta, 2009). For example, tropical ectotherms have been postulated to have a higher risk of extinction due to climate warming as they are more likely to have evolved as thermal specialists, increasing their sensitivity to fluctuations in temperature (Deutsch et al., 2008; Tewksbury, Huey, & Deutsch, 2008; but see Walters, Blanckenhorn, & Berger, 2012).

Thermal sensitivity can be presented as a thermal performance curve (TPC): a usually a left‐skewed Gaussian distribution limited by critical minimum (CT_min_) and maximum (CT_max_) temperatures (Angilletta, 2009; Huey & Kingsolver, 1989). CT_max_ allows estimation of an organisms’ “warming tolerance”, defined as the difference between CT_max_ and average temperature of the natural habitat (*T*_hab_; Deutsch et al., 2008). The performance is maximized at optimal temperature (*T*_opt_), which is of high interest when the trait is fitness‐related and putatively under selection. In addition, *T*_opt_ is used to estimate the “thermal safety margin” (TSM; defined as the difference between *T*_opt_ and *T*_hab_). However, as different traits often have different *T*_opt_, estimating an overall *T*_opt_ can be difficult (Angilletta, 2009; Huey & Stevenson, 1979; Martin & Huey, 2008). As ectotherms use thermoregulatory behavior as a mean to maximize their performance (Grant & Dunham, 1988; Martin & Huey, 2008), the preferred temperature (*T*_p_) is often used as a proxy for the overall *T*_opt_ (Angilletta, 2009).

Thermal performance evolves in response to variation in thermal regimes (Angilletta, 2009). For example, studies over larger geographic scales have shown that both CT_max_ and CT_min_ decrease with increasing latitude (Addo‐Bediako, Chown, & Gaston, 2000; Sunday, Bates, & Dulvy, 2011), but CT_max_ appears to be evolutionarily more rigid than CT_min_ (Araújo et al., 2013). However, thermal tolerance can be affected by both intensity and duration of a thermal stress. Recently, Rezende, Casteneda, and Santos (2014) suggested that there may be a trade‐off between acute and chronic tolerance of high temperatures. This trade‐off may explain the counter‐intuitive results where warm‐origin populations have lower CT_max_ than cold‐origin populations (e.g., Castañeda, Rezente, & Santos, 2015; Sgrò et al., 2010).

A common assumption in optimality models of thermal adaptation (e.g., Gilchrist, 1995; Lynch & Gabriel, 1987) is that there is a close match between an organism's *T*_p_ and *T*_opt_ (Angilletta, 2009). However, in organisms that are poor thermoregulators, *T*_p_ should be lower than *T*_opt_ (Martin & Huey, 2008). This is also true in situations when locally adapted individuals from contrasting environments differ in their performance (Asbury & Angilletta, 2010). Such differences could arise when there are differences in food availability or when maximum performance increases with optimum temperature (i.e., “Hotter is better”; Bennett, 1987; Kingsolver & Huey, 2008; Angilletta, 2009). Any mismatch between *T*_p_ and *T*_opt_ is predicted to increase with temperature variation within generations (Asbury & Angilletta, 2010; Lynch & Gabriel, 1987). Alternatively, although thermal specialists generally evolve in environments with low temperature variation (Ghalambor, Huey, Martin, Tewksbury, & Wang, 2006; Janzen, 1967), they are expected to show a larger difference between *T*_p_ and *T*_opt_ than thermal generalists, because the increased asymmetry of the TPC increases their sensitivity to temperature fluctuations (Martin & Huey, 2008).

Importantly, while studies on thermal performance over larger geographic gradients (reviewed by e.g., Addo‐Bediako et al., 2000; Araújo et al., 2013; Sunday et al., 2011) have increased our understanding of large‐scale effects of climatic variation and warming, they are less helpful in understanding how closely located populations will evolve in response to local variation in temperatures. Specifically, gene flow can have a strong effect on local adaptation over short spatial distances (Kawecki & Ebert, 2004; Räsänen & Hendry, 2008; Sultan & Spencer, 2002) and influence how populations adapt to climate change (de Mazancourt, Johnson, & Barraclough, 2008). In order to better understand how thermal adaptation is affected by the combined effects of gene flow and natural selection, studies of systems allowing gene exchange over shorter distances are needed (Keller, Alexander, Holderegger, & Edwards, 2013; Logan, Dureya, Molnar, Kessler, & Calsbeek, 2016; Merilä & Hendry, 2014; Richter‐Boix et al., 2015).

**Figure 1:**
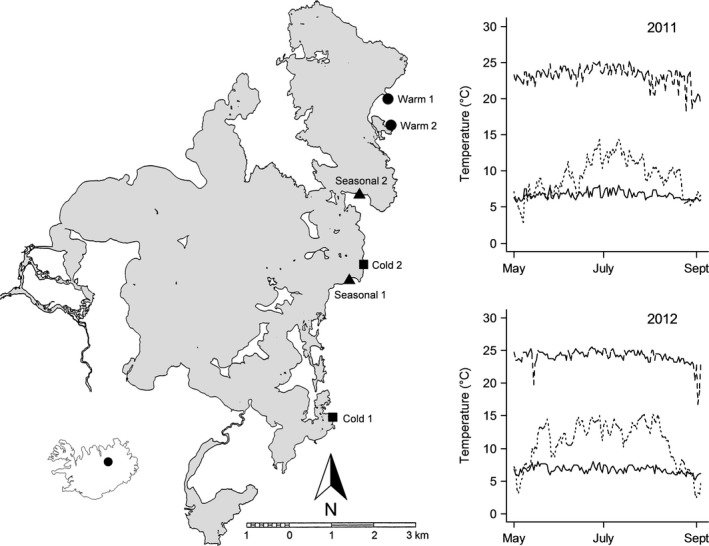
Map of Lake Mývatn and the locations of the sampled thermal habitats. The figures indicate temperature measurements from the three thermal environment types during May 18 and September 18, 2011 (above) and 2012 (below). Cold, seasonal, and warm are drawn with solid, dotted, and dashed lines, respectively

Here we investigated thermal preference (*T*_p_) and maximum temperature tolerance (CT_max_) in the common pond snail *Radix balthica* originating from three contrasting thermal environments within Lake Mývatn, northern Iceland. In Lake Mývatn, geothermally heated groundwater creates a system of cold (ca. 7°C) and warm (ca. 23°C) springs along the shore line (Figure 1). While temperature variation close to the springs is low, these habitats are connected by areas with seasonal temperature variation (Figure 1). Genetic studies show that populations in similar thermal habitats are significantly differentiated in neutral molecular markers, but they are more similar in markers putatively under selection (Johansson, Quintela, & Laurila, 2016; Quintela, Johansson, Kristjansson, Barreiro, & Laurila, 2014). Yet these very contrasting thermal environments are connected by low gene flow (Johansson, Quintela, et al., 2016; Quintela et al., 2014). Hence, Lake Mývatn constitutes an ideal system to evaluate the evolution of *T*_p_ and CT_max_ in the presence of potentially disrupting evolutionary forces.

As a small aquatic ectotherm with low mobility, *R*. *balthica* is a poor thermoregulator with body temperature equalling the surrounding environment (Reynolds & Casterlin, 1979). Although snails in the close vicinity of the geothermal springs can be expected to show a higher degree of thermal specialization than snails in habitats subjected to seasonal temperature variation (Ghalambor et al., 2006; Janzen, 1967), laboratory and field experiments showed that life history patterns were better explained by cogradient variation (Conover, Duffy, & Hice, 2009) with warm‐origin snails have higher growth and reproduction in all temperatures (Johansson, Ermold, Kristjansson, & Laurila, 2016). However, snails from all habitats had roughly similar *T*_opt_ for growth and reproduction (Johansson, Ermold, et al., 2016). As these two traits are important fitness components, we expect to find a common *T*_p_ irrespective of thermal origin. An alternative prediction is that seasonal origin snails have a lower *T*_p_ than either cold or warm‐origin snails due to the higher variation in environmental temperature requiring a larger overall TSM. Previous common garden and reciprocal transplant experiments showed that warm‐origin snails had higher survival in high temperatures (Johansson, Ermold, et al., 2016). Consequently, we expect to find the highest CT_max_ in warm‐origin populations and that seasonal origin snails are more similar to cold origin snails, as seasonal habitats have temperatures closer to those experienced in the temporally stable cold sites. Alternatively, if acute thermal stress tolerance trades off with chronic stress tolerance (Rezende et al., 2014), we expect that warm‐origin snails have lowest CT_max_.

## 2 Methods

*Radix balthica* is a pulmonate snail common in freshwaters throughout northern Europe (Lakowitz, Brönmark, & Nyström, 2008; Pfenninger, Salinger, Haun, & Feldmeyer, 2011) and the most common gastropod in Lake Mývatn. Although it has direct development, it is often considered an early colonizer mainly relying on bird‐assisted dispersal (Haun, Salinger, Pachzelt, & Pfenninger, 2012; Lakowitz et al., 2008; Pfenninger et al., 2011).

Lake Mývatn (65°60′N, 17°00′W; Figure 1) was formed in a lava eruption ca. 2,300 years ago (Einarsson et al., 2004). It is fed by ground water that passes through differently heated volcanic bedrock, forming cold springs in the south‐eastern part of the lake and warm springs in the north‐eastern part (Einarsson et al., 2004; Ólafsson, 1979). Temperature close to the springs is relatively stable over time, although small temperature fluctuations occur due to variation in geothermal activity (Ólafsson, 1979; Figure 1). The area affected by the springs is extensive as indicated by the fact that a large proportion of the eastern shore of Lake Mývatn remains ice‐free during winter (Ólafsson, 1979). Field measurements of temperature were taken between early May and late September 2011 and 2012 with iButton^®^ DS1921G temperature loggers programmed to record temperature every fourth hour. Temperature is reported as the average from three temperature loggers placed at the bottom at 50 cm depth at each site. Two of the six sites selected for this study (Figure 1) were subjected to seasonal temperature variation (mean °C ± *SE*, Seasonal 1: 8.59 ± 0.06, Seasonal 2: 9.13 ± 0.07) and the remaining four were classified as either temporally stable cold (Cold 1: 6.39 ± 0.02, Cold 2: 7.52 ± 0.02) or warm (Warm 1: 22.96 ± 0.04, Warm 2: 22.20 ± 0.03).

We collected 100–150 juvenile *R*. *balthica* (shell length 5–8 mm) at each of the six sites in June 2011 (Figure 1). Previous studies showed that snails in all these sites (henceforth populations) were significantly genetically differentiated from each other (even within a thermal habitat‐type) in neutral AFLP‐markers (Johansson, Quintela, et al., 2016). The snails were transported to Uppsala University where they were maintained in a walk‐in climate‐controlled room at 12°C with a 16 hr light:8 hr dark regime. Each population was kept in multiple 35 × 25 × 25 cm plastic aquaria filled with 15 L of reconstituted soft water (RSW; APHA, 1985). The snails were allowed to reproduce freely within each aquarium and all egg batches were collected and placed in new aquaria. The experiments were conducted on the F1 generation. Importantly, as the experiments were conducted on the first laboratory generation, we cannot exclude the possibility that our results are influenced by cross‐generational effects (Räsänen & Kruuk, 2007). A common garden experiment conducted on laboratory reared F3 snails estimated a common *T*_opt_ at 20°C for growth rate and at 16°C for reproduction (Johansson, Ermold, et al., 2016). We used the first temperature as a measure of *T*_opt_ throughout the manuscript as growth has a major influence on ectotherm life history and size is a good predictor of fecundity in pulmonates (Dillon, 2010; Kingsolver & Huey, 2008).

### 2.1 Preferred temperature (*T*_p_)

In order to create an experimental temperature gradient, ten 500 × 20 × 20 mm channels separated by 2 mm were milled in a single block of aluminium. Each channel was filled with 125 ml RSW. The block was positioned so that one end rested on a warming plate and the other end on a cooling plate, creating a temperature gradient with a difference of 24°C (min: 1 ± 0.5°C, max: 25 ± 1°C). The temperature gradient was stable between trials (adj‐R^2^ = 0.95) with an average temperature increase of 0.36°C/cm. Twenty snails (average shell length 7.4 ± 0.9 [*SE*] mm) were randomly selected from each site and placed individually at the 12°C mark in a randomly assigned channel. Temperature at the location of each snail was measured with a digital thermometer (±1°C) every 30 min for 6 hr. The average of three temperature readings at each time point was used in the analyses. Temperature preference was defined as the average of temperature measurements during the last 4 hr as the first 2 hr often are influenced by the acclimation temperature (Reynolds & Casterlin, 1979). To determine if the snails had chosen a temperature, the distribution of preferred temperatures was compared to a random distribution based on an equal sample size assuming that there were no differences in movement speed between the populations (Dillon, Liu, Wang, & Huey, 2012). After the trial the snails were placed individually in plastic vials with 100 ml RSW at 20°C.

### 2.2 Maximum critical temperature (CT_max_)

CT_max,_ defined as the temperature, at which the snail is unable to maintain attachment to a surface (Díaz et al., 2006; Sandison, 1967), was measured 18 hr after the snails were subjected to the temperature gradient. The snails were placed individually in a 50 ml Falcon tubes completely filled with RSW. The tubes were placed in a water bath at 20°C and temperature was increased by 1°C every 15 min (Díaz, Salas, Re, Gonzalez, & Reyes, 2011; Díaz et al., 2006) until the snail lost attachment. Thereafter, the snails were immediately placed at room temperature and left to recover for 24 hr. The recovery success after the thermal challenge was very high (over 90% in all populations) and no statistical tests on the recovery data were conducted.

### 2.3 Statistical analyses

All statistical analyses were performed in R (R Core Team, 2013). Model assumptions were checked following Zuur, Ieno, and Elphick (2010). Individual preferred temperatures were analyzed with an ANOVA using thermal origin as a factor. Sites within thermal origin were pooled as there were no differences in *T*_p_ between sites (*t* tests, *p* > .38). Snail size (shell length) was not correlated with *T*_p_ (*r*_117_ = −.05, *p* = .58).

Differences in CT_max_ between thermal origins were analyzed with an ANOVA with site pooled within thermal origin (*t* tests, *p* > .14). Pearson correlations between CT_max_ and *T*_p_ were calculated both over all thermal origins and within each thermal origin. Only snails that had recovered 24 hr after losing attachment were included in the analyses on CT_max_; however, the results did not change qualitatively when all snails were included. In addition to CT_max_, we estimated the temperature at which half of the snails remained attached (CT_50_) for each thermal origin. This was made by fitting a curve with the R‐function “loess” to the data points representing the percentage of attached snails at each temperature.

## 3 Results

**Figure 2:**
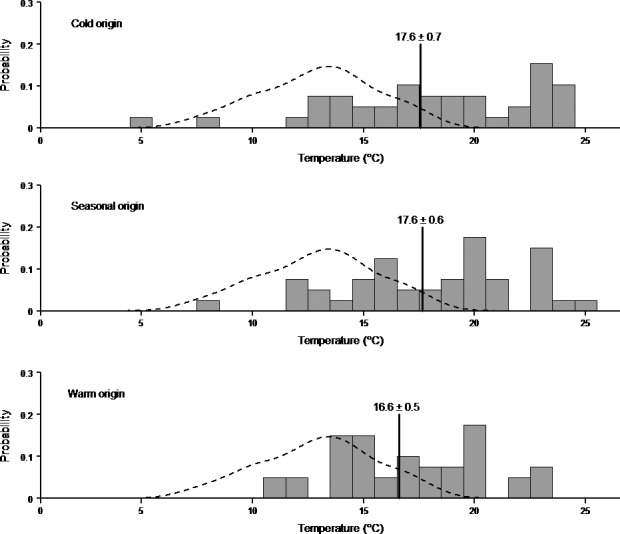
Probability histograms of average individual preferred temperature (°C) for each of the thermal origins. The dashed line represents the distribution of averages if the snails had moved randomly in the temperature gradient. The vertical black line is the mean (±*SE*) preferred temperature

The preferred temperature was different from a random distribution in all thermal origins (Cold: *t*_38_ = 7.51, *p* < .0001, Seasonal: *t*_39_ = 8.93, *p* < .0001, Warm: *t*_39_ = 8.75, *p* < .0001; Figure 2). There was no difference in preferred temperature (ca. 17°C) between thermal origins (*F*_2,116_ = 0.87, *p* = .42; Figure 2). There was also no difference in the highest visited individual temperature between the thermal origins (*F*_2,116_ = 1.21, *p* = .30) in the preferred temperature trials. However, the lowest visited individual temperature for cold origin snails was higher than for seasonal or warm‐origin snails (*F*_2,116_ = 3.90, *p* = .02) and, consequently, cold origin snails visited a significantly narrower range of temperatures than other snails (*F*_2,116_ = 6.28, *p* < .01).

**Figure 3:**
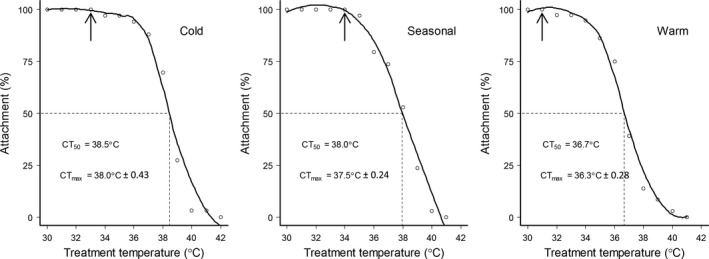
Percentage of *Radix balthica* remaining attached plotted against temperature. CT_50_ (dashed line) is calculated based on the fitted polynomial (solid line). CT_max_ (±*SE*) and CT_50_ are shown in the figure. Arrows indicate where snails start to lose attachment

Snails from cold, seasonal, and warm thermal origins started to loose attachment at 33, 34, and 31°C, respectively (Figure 3). Warm‐origin snails had a significantly lower CT_max_ and CT_50_ than either cold or seasonal origin snails (*F*_2,100_ = 11.13, *p* < .001; Figure 3). However, there was no difference in CT_max_ or CT_50_ between cold and seasonal origin snails (Figure 3). There was an overall positive correlation between CT_max_ and thermal preference (*r*_101_ = .25, *p* < .001), as well as among warm‐origin snails (*r*_34_ = .37, *p* = .026). However, there was no significant correlation between *T*_p_ and CT_max_ within cold (*r*_32_ = .10, *p* = .57) or seasonal (*r*_31_ = .25, *p* = .16) origin snails.

## 4 Discussion

Both cold and warm habitat snails experienced environments of low temperature variation, although the mean temperatures differed strongly (Figure 1). Thermal variation was much larger in seasonal habitats, while the mean temperature was closer to that in the cold habitats. Despite these large temperature differences between their native habitats, we found that *R*. *balthica* from different thermal origins had a common *T*_p_. This was in accordance with our prediction based on the common *T*_opt_ between the populations. Surprisingly, we found that warm‐origin snails, inhabiting environments with 15°C higher mean temperature than cold and seasonal origin snails, had lower CT_max_ than cold and seasonal populations. As high CT_max_ is generally positively correlated with environmental temperature in aquatic organisms (Sunday et al., 2011), this result seems counterintuitive. However, as our earlier studies have shown that under chronic exposure to high temperatures warm‐origin snails have higher survival—both in the field and in the laboratory—than cold and seasonal snails (Johansson, Ermold, et al., 2016), these results agree with the recent analyses by Rezende et al. (2014) showing that CT_max_ of an organism is expected to decrease with the duration of the thermal challenge, warm‐origin snails trading off CT_max_ with chronic stress tolerance. Accumulating evidence suggests that this pattern may be common among ectotherms, examples including studies on *Drosophila* across large geographic gradients (Castañeda et al., 2015; Sgrò et al., 2010), as well as interspecific comparisons in reptiles (Araújo et al., 2013; Huey et al., 2009). To our knowledge, this is the first study to demonstrate this pattern in network of closely located populations.

The physiological mechanisms affecting the trade‐off between acute and thermal tolerance are unknown. As discussed by Rezende et al. (2014), many biochemical and physiological processes (e.g., enzyme denaturation, neural function, oxygen limitation, metabolic balance, and immunity) are expected to be affected differentially by different forms of thermal stress. In general, warm‐origin snails inhabit a thermal environment associated with constant costs of thermal adaptation, whereas cold and seasonal origin snails may rely more on plastic responses, including production of heat shock proteins (Hsp) in response to acute temperature stress (Narum, Campbell, Meyer, Miller, & Hardy, 2013; Sørensen, Kristensen, & Loeschcke, 2003). However, high Hsp levels over an extended time can be detrimental, and warm‐origin snails may rely on constant but lower levels of Hsp expression enabling them to manage the warm environment in the long‐term (Narum et al., 2013; Sørensen et al., 2003). Indeed, the risk of experiencing temperatures close to the estimated CT_max_ is nonexistent as temperatures at warm sites are stable over time and temperature fluctuations at the warm sites only reduce the water temperature (e.g., cooler surface water coming in during a storm). Importantly, temperatures never reach values as high as above 31°C when warm‐origin snails start to loose attachment to the surface. In line with this hypothesis, a study comparing lake chubs (*Couesius plumbeus*) from contrasting geothermal habitats found that populations from temporally stable warm habitats had lower CT_max_ than those from temporally varying warm habitats (Darveau, Taylor, & Schulte, 2012).

Interestingly, the cold and seasonal populations have maintained the higher CT_max_, although even the seasonal populations very rarely meet temperatures over 20°C in their natural habitats. This suggests that there is very little selection for reduced CT_max_ in the cold and seasonal habitats, in line with the general evolutionary rigidness of this trait (Araújo et al., 2013). This reasoning also suggests the trade‐off between high CT_max_ and fitness in the warm habitat is strong. The significant positive correlation between *T*_p_ and CT_max_ found in warm‐origin snails, but not in seasonal or cold origin snails, may further suggest stronger thermal selection in the warm environment. The positive correlation may suggest that selection to maintain the warming tolerance (CT_max_ − *T*_hab_) at the individual level is stronger in the warm than in the seasonal or cold habitats, but more studies are clearly needed.

As thermal populations in Lake Mývatn are connected by some gene flow (Johansson, Ermold, et al., 2016; Quintela et al., 2014), the fact that CT_max_ differs between contrasting thermal habitats in Lake Mývatn suggests that the thermal characteristics of the environment are a stronger selective factor than the potentially homogenizing effect of gene flow. Seasonal habitats are the ancestral environment, and in the warm habitats the ancestral seasonal origin snails have evolved improved chronic thermal tolerance and lower CT_max_, while retaining a common *T*_p_. This scenario is supported by the fact that *T*_opt_ rarely diverges in the presence of gene flow (Angilletta, 2009; but see Logan et al., 2016; Richter‐Boix et al., 2015), but tolerance limits can evolve rapidly in response to changes in environmental temperature (especially CT_min_; Barrett et al., 2011; Geerts et al., 2015; Leal & Gunderson, 2012; Logan, Cox, & Calsbeek, 2014). Theoretical work predicts that evolution of *T*_p_ in the presence of gene flow is dependent on the environmental asymmetry of the habitats, infrequent migration favoring a *T*_p_ closer to the mean temperature of the better environment (Angilletta, 2009; Day, 2000). Assuming that the warm environment is of higher quality than the cold environment (i.e., “hotter is better”), the common overall *T*_p_ closer to the temperature in the warm habitats suggests that the level of gene flow is relatively low (Angilletta, 2009; Day, 2000). This is in accordance with the significant population structure we have found among the present populations (Johansson, Quintela, et al., 2016).

The discrepancy between *T*_p_ and *T*_opt_ is expected to increase both with temperature variation as well as with the level of thermal specialization in order to buffer against the reduction in performance at temperatures above the optimum (Martin & Huey, 2008). The relative strengths of these factors (i.e., level of thermal specialization and temperature variation) can in principle produce a similar *T*_p_ despite different TPCs. However, as empirical data suggest that strict thermal specialists (i.e., with very narrow thermal optima) are rare (Angilletta, 2009; Conover et al., 2009), and as we found little evidence of strict thermal specialization in snails originating from the thermal environments of Lake Mývatn (apart from survival in the cold and warm environments; Johansson, Ermold, et al., 2016), we find this scenario unlikely. Due to the high impact of size on life‐history traits in ectotherms we suggest that the common *T*_p_ is most likely due to common *T*_opt_ for growth and reproduction.

Cold origin snails were similar to seasonal snails in terms of *T*_p_ and CT_max_, but moved within a narrower range of temperatures during the last 4 hr in the temperature gradient. This was because cold‐origin snails avoided the lowest temperatures more than seasonal or warm‐origin snails, while there was no difference in the maximum temperature visited. Although seasonal origin snails can experience temperatures above 15°C in nature, cold origin snails do not. However, also cold‐origin snails benefitted from higher temperatures in terms of higher growth and fecundity in laboratory and field experiments (Johansson, Ermold, et al., 2016). Interestingly, our results suggest that when encountering a favorable temperature around 16–20°C, cold origin snails seem to be more exact thermoregulators and better at utilizing the beneficial thermal environment.

To summarize, our results suggest that the contrasting thermal environments in Lake Mývatn have selected for divergent thermal adaptations in *R*. *balthica*. Specifically, as there was no difference in tolerance between cold and seasonal origin snails, our results point to the effect of high mean temperatures rather than temperature variation per se (Kingsolver, Ragland, & Diamond, 2009; Ragland & Kingsolver, 2008). At the scale of a single lake, our results support the concept of thermal tolerance landscapes (Rezende et al., 2014), where warm‐origin snails have traded off tolerance of acute thermal stress in favor of higher performance at the relatively high temperatures they experience in their natural habitat. While increasing the sensitivity to extreme temperatures, higher performance in chronically high temperature environment has been suggested to buffer against changing temperatures and is one of the processes through which warm adapted organisms can cope with climate change (Gomulkiewicz & Holt, 1995; Lynch & Lande, 1993; Walters et al., 2012). Finally, our results support the notion that in order to get a more comprehensive understanding of thermal adaptation several measures of tolerance are needed.

## Conflict of Interest

None declared.
